# Human Melanoma Cells Differentially Express RNASEL/RNase-L and miR-146a-5p under Sex Hormonal Stimulation

**DOI:** 10.3390/cimb44100326

**Published:** 2022-10-11

**Authors:** Elisa Orlandi, Elisa De Tomi, Rachele Campagnari, Francesca Belpinati, Monica Rodolfo, Elisabetta Vergani, Giovanni Malerba, Macarena Gomez-Lira, Marta Menegazzi, Maria Grazia Romanelli

**Affiliations:** 1 Section of Biology and Genetics, Department of Neurosciences, Biomedicine and Movement Sciences, University of Verona, Strada Le Grazie, 8, 37134 Verona, Italy; 2 Section of Biochemistry, Department of Neurosciences, Biomedicine and Movement Sciences, University of Verona, Strada Le Grazie, 8, 37134 Verona, Italy; 3 Unit of Immunotherapy of Human Tumors, Department of Research, Fondazione IRCCS Istituto Nazionale dei Tumori, 20133 Milan, Italy

**Keywords:** mRNA/miR-146a interaction, testosterone, 17β-estradiol, melanoma cells, gene regulation

## Abstract

Polymorphisms in the ribonuclease L (RNASEL) coding gene and hsa-miR-146a-5p (miR-146a) have been associated with melanoma in a sex-specific manner. We hypothesized that RNASEL and miR-146a expression could be influenced by sex hormones playing a role in the female advantages observed in melanoma incidence and survival. Thus, we explored the effects of testosterone and 17β-estradiol on RNASEL and miR-146a expression in LM-20 and A375 melanoma cell lines. Direct targeting of miR-146a to the 3′ untranslated region (3′UTR) of RNASEL was examined using a luciferase reporter system. Our results indicate that RNASEL is a direct target of miR-146a in both melanoma cell lines. Trough qPCR and western blot analyses, we explored the effect of miR-146a mimic transfection in the presence of each hormone either on RNASEL mRNA level or on protein expression of RNase-L, the enzyme codified by RNASEL gene. In the presence of testosterone or 17β-estradiol, miR-146a overexpression did not influence RNASEL transcript level in LM-20 cell line, but it slightly induced RNASEL mRNA level in A375 cells. Remarkably, miR-146a overexpression was able to repress the protein level of RNase-L in both LM-20 and A375 cells in the presence of each hormone, as well as to elicit high expression levels of the activated form of the extracellular signal-regulated kinases (ERK)1/2, hence confirming the pro-tumorigenic role of miR-146a overexpression in melanoma. Thereafter, we assessed if the administration of each hormone could affect the endogenous expression of RNASEL and miR-146a genes in LM-20 and A375 cell lines. Testosterone exerted no significant effect on RNASEL gene expression in both cell lines, while 17β-estradiol enhanced RNASEL transcript level at least in LM-20 melanoma cells. Conversely, miR-146a transcript augmented only in the presence of testosterone in either melanoma cell line. Importantly, each hormone acted quite the opposite regarding the RNase-L protein expression, i.e., testosterone significantly decreased RNase-L expression, whereas 17β-estradiol increased it. Overall, the data show that, in melanoma cells treated with 17β-estradiol, RNase-L expression increased likely by transcriptional induction of its gene. Testosterone, instead, decreased RNase-L expression in melanoma cell lines with a post-transcriptional mechanism in which miR-146a could play a role. In conclusion, the pro-tumor activity of androgen hormone in melanoma cells could be exacerbated by both miR-146a increase and RNase-L downregulation. These events may contribute to the worse outcome in male melanoma patients.

## 1. Introduction

Melanoma incidence is continuously increasing in most developed countries [1]; epidemiologic data suggest that besides UV exposure, other factors such as sex, genetics, and epigenetics enhance the risk of developing cutaneous melanoma [2]. Sex differences in melanoma incidence and outcome have been constantly registered, yet not completely explained [3,4]. Male gender is indeed associated with a greater incidence of primary melanoma, and gender is an independent factor affecting survival in melanoma patients [5]. Complex molecular mechanisms and several exogenous and endogenous factors are involved in melanoma progression. One of which is the sex hormones milieu, which can influence gene expression by acting at both transcriptional and post-transcriptional levels [6,7,8].

Recently, melanoma has been reported to grow faster in male than in female mice [9]. Testosterone could likely be responsible for the worse melanoma prognosis in males. Indeed, it can induce melanoma cell growth via androgen receptor (AR) since genetic and pharmacological suppression of AR activity in melanoma cells blunts their proliferation, while increased AR expression or activation exerts opposite effects [10]. Remarkably, AR expression is positively correlated with poor survival of cutaneous melanoma patients [11]. AR, indeed, plays a key role in increasing melanoma cell invasion in multiple cell lines in vitro and in a mouse model in vivo [11]. Nevertheless, other authors claimed that testosterone supported melanoma cell growth also independently of AR binding, i.e., by inducing zinc influx and activating Mitogen Activating Protein Kinases (MAPK) [9]. At the same time, the melanoma protective effect in females could result from estrogen signaling through the G protein-coupled estrogen receptor (GPER) [12]. GPER activation in melanoma induces several phenotypic changes that inhibit tumor growth, and also render tumor cells more susceptible to clearance by native immune cells [12].

We previously observed a sex-specific interaction between rs486907 polymorphism in the ribonuclease L (RNASEL) gene and rs2910164 in miR-146a gene, showing that, only in the male population, allele rs2910164C represents a risk factor for individuals carrying the RNASEL rs486907GG genotype [13]. This sex-dependent linkage of both genes has been observed in non-melanoma skin cancer, as well [14]. Additionally, the same miR-146a polymorphism has been associated with various diseases in a sex-dependent fashion [15,16,17]. Micro RNA (miRNAs) are non-coding RNAs able to inhibit translation or induce mRNAs degradation by binding to their mRNA targets in specific complementary sequences usually located in the 3′ untranslated regions 3′(UTR), although they can bind also to other mRNA sequences, such as the 5′UTR or the coding region [18]. MiRNAs can be sex- and tissues-differentially expressed [19,20], suggesting their implication in sex bias in disease predisposition or outcome. Importantly, the different miRNAs expression level is a common attribute of malignant cell phenotype compared to normal one [21,22]. It should be noted that miR-146a is an important modulator of inflammation by affecting the innate and adaptive immune response [23]. Pan et al. reported that macrovesicles containing high level of miR-146a significantly promoted microvascular endothelial cell proliferation with the associated increase of the extracellular signal-regulated kinases (ERK)1/2 phosphorylation level [24]. In cancer, miR-146a can have either tumor promotive or tumor suppressive effects [25], although in melanoma it plays an oncogenic role [26].

RNASEL gene encodes the 2′,5′-oligoadenylate synthetase-dependent ribonuclease L (RNase-L), an enzyme that displays an antiviral role and may control the half-life of several mRNAs [27]. RNASEL/RNase-L regulate critical cellular functions including host antiviral response, apoptosis, and tumor-suppressive activity; thus, their expression must be tightly regulated [28,29,30]. The contribution of the RNASEL 3′UTR to the stability of its mRNA has been previously investigated [31]. Apart from several AU rich elements, RNASEL 3′UTR contains potential binding sites for some miRNAs, including a putative binding site for miR-146a [31].

In the present work, firstly we assessed whether RNASEL mRNA could be a direct target of miR-146a in LM-20 and A375 melanoma cells and in HaCaT cells. HaCaT is a human adult, low-calcium, high-temperature, keratinocyte cell line, which represents a cellular model commonly used to study normal keratinocytes [32,33,34]. The cells exhibit, similar to human keratinocytes, the following features: physiological keratinocyte morphology, epidermal differentiation capability, non-tumorigenic, and can undergo ultraviolet light-induced apoptosis [35]. Keratinocytes, indeed, can impact melanoma progression as they belong to tumor microenvironment by closely interacting with melanocytes in skin melanoma.

Again, we investigated whether the transfection of miR-146a mimic in the presence of each sex hormone could affect the RNASEL/RNase-L expression level, as well as ERK1/2 activity (pERK1/2).

Finally, we measured the endogenous levels of miR-146a and RNASEL/RNase-L expression in melanoma cells treated with either testosterone or 17β-estradiol.

Our results suggest that sex hormones can influence RNase-L expression, i.e., testosterone decreased it by a post-transcriptional mechanism involving the increment of miR-146a gene expression, whereas 17β-estradiol was able to enhance RNase-L level by inducing RNASEL transcription.

## 2. Materials and Methods

### 2.1. Cell Culture

Melanoma cell line LM-20 (17697M) derived from a male nodal metastasis [36] was provided by Dr Monica Rodolfo (Istituto Nazionale Tumori, Milan). A375 melanoma cells (CRL-1619; ATCC, Manassas, VA, USA) and LM-20 cells were grown in Roswell Park Memorial Institute 1640 (RPMI-1640, Gibco, BTL, Invitrogen Corp., Carlsbad, CA, USA).

HaCaT cells were originally isolated from human adult skin and named based on their origin (human, adult, low-calcium, high-temperature, keratinocytes) [35]. HaCaT is considered a permanent non-malignant epithelial cell line, which maintains full epidermal differentiation capability [35]. Although it exhibits spontaneous phenotypic transformation and despite the unlimited growth potential, HaCaT cells, similar to normal keratinocytes, it reforms an orderly structured and differentiated epidermal tissue when transplanted onto nude mice, remaining non-tumorigenic [32]. Keratinocytes HaCaT cells (CLS, Cell Lines Service, Eppelheim, Germany) were grown in Dulbecco’s modified Eagle’s medium (DMEM, Gibco, BTL, Invitrogen Corp. Carlsbad, CA, USA) containing 4.5 g/L D-glucose. All media were supplemented with 10% heat-inactivated fetal bovine serum, 1% L-glutamine (200 nM solution) and 2% Penicillin-Streptomycin (5000 I.U/mL and 5000 µg/mL solution, respectively) all purchased from Gibco, BTL, Invitrogen Corp. Carlsbad, CA, USA. All cell cultures were maintained in an incubator in a humidified atmosphere, of 5% CO_2_, at 37 °C.

### 2.2. Cloning of the RNase-L 3′UTR, Generation of 3′UTR Mutants and Transfections

A 777bp fragment of the RNASEL 3′UTR (NM_021133.4) was amplified using primers described in Table 1, which introduce an Xba I restriction site at the 5′ and 3′ of the amplified fragment.

The amplified fragment was subcloned downstream of the firefly luciferase gene in the pGL3 promoter vector (3′UTR_RNASEL_WT). A 3′UTR_RNASEL_WT plasmid containing 5 nucleotide deletion in the consensus site for miR-146a was obtained by QuikChange Site-Directed Mutagenesis Kit (Stratagene, Agilent Technologies, La Jolla, CA, USA) (3′UTR_RNASEL_MUT). Primers used for amplification and mutagenesis are reported in Table 1. The plasmid sequences were confirmed through restriction enzyme mapping analysis and DNA sequencing (BMR Genomics, Padova, Italy).

Transient transfection of the recombined vectors was performed using Lipofectamine 3000 (Invitrogen Corp., Carlsbad, CA, USA), according to the manufacturer’s instructions. The two melanoma cell lines and HaCaT keratinocytes (1.3 × 10^5^ cells) were seeded in 24 well plates. After 24 h, 1 µg of 3′UTR_RNASEL_WT or 1 µg of 3′UTR_RNASEL_MUT constructs, together with 50 nM miR-146a mimic and 5 ng of *Renilla* luciferase plasmid (as normalizer) were transfected. The pGL3 vector without insert was used as a negative control. After 24 h, the cells were lysed with Passive Lysis Buffer (Promega Italia srl, Milan, Italy), and the relative luciferase activity was assessed with the Dual-Luciferase Assay Reporter System (Promega Italia srl, Milan, Italy) [37].

### 2.3. Cell Treatment with Testosterone or 17β-Estradiol

For qPCR, cells were plated at 3 × 10^5^ cells per well in a 6 well plate in 2 mL of RPMI phenol red-free supplemented with 10% charcoal treated serum, 1% L-glutamine (200 nM solution) and 2% Penicillin-Streptomycin (5000 I.U/mL and 5000 µg/mL solution, respectively) for 48 h. For western blot, seven thousand five hundred (7.5 × 10^5^) cells were seeded in T25 flasks and incubated in 5 mL of RPMI phenol red-free supplemented with 10% charcoal treated serum, and 1% L-glutamine (200 nM solution) and 2% Penicillin-Streptomycin (5000 I.U/mL and 5000 µg/mL solution, respectively). Next, testosterone or 17β-estradiol (Sigma Aldrich, Milan, Italy), at the final concentration of 10^−6^ M and 5 × 10^−7^ M, respectively, was added to the medium and cells were cultured for the following 24 h. Cells incubated with the corresponding amounts of pure ethanol (<0.0001%) served as a negative control and were set to 1 for analysis. Hormone concentrations were chosen based on experiments described by Kanda et al. [38] and after cell viability testing. Cells were subsequently lysed with Trizol Reagent (Thermo Fisher Scientific, Milano, Italy) for RNA extraction or RIPA buffer for western blot (see below).

#### 2.3.1. RNA Extraction, Reverse Transcription and Real-Time PCR

Total RNA for gene expression analyses was extracted with Trizol Reagent (Thermo Fisher Scientific, Milan, Italy), following the manufacturer’s protocol. RNA quantification was performed with Nanodrop 2000 spectrophotometer (Thermo Fischer Scientific, Milan, Italy), and 500 ng of RNA were retrotranscribed by SensiFAST cDNA Synthesis Kit (Bioline, Trento, Italy), following the manufacturer’s protocol.

For miRs expression analyses, 1 µL for each sample of the total RNA was direct reverse transcribed with the TaqMan Advanced miRNA cDNA Synthesis Kit (Thermo Fisher Scientific, Milan, Italy), following the manufacturer’s protocol.

After reverse transcription, RNASEL and miR-146a expression levels were determined by real-time polymerase chain reaction (qPCR) as described [39]. Normalization was performed using the TATA box protein (TBP) which showed to be the most stable gene in our experimental conditions [40]. Primers used for amplification are described in Table 1.

For miRNAs analysis, TaqMan Fast Advanced Master Mix and the specific probe for miR-146a (Thermo Fisher Scientific, Milan, Italy) were used. Normalization of expression was performed by miR-191, which showed to be the most stable miRNA in our experimental conditions [40].

The Real-Time PCR was performed in the Bio-Rad CFX Connect Real-Time System using the SensiFAST SYBR no-rox Kit (Bioline, Trento, Italy) or the TaqMan Advanced microRNA probes (Thermo Fisher Scientific, Milan, Italy). Relative quantification was calculated by Pfaffl’s formula [41]. Each measurement was carried out in triplicate in at least three different experiments.

#### 2.3.2. Western Blot Analysis

After incubation with hormones, cells were washed twice with ice-cold Phosphate-buffer solution (PBS) and then collected by scraping. Subsequent protein extraction and western blotting were performed essentially as previously described [42]. Briefly, cells were lysed in RIPA buffer and a mixture of protease inhibitors (Mirus Bio LLC, Medison, WL, USA). Total protein concentration was determined by detergent compatible (DC) Bradford Assay analysis (Thermo Fisher Scientific, Milan, Italy). Cellular proteins were separated in SDS polyacrylamide 10% gel electrophoresis (SDS-PAGE) and transferred onto PVDF membrane (Thermo Fisher Scientific, Milan, Italy) by a wet electrophoretic transfer method. Membranes were incubated for 2 h at room temperature with primary antibodies diluted in blocking buffer: the anti-RNase-L (1:10,000, Wuhan Fine Biotech Co, Dublin, CA, USA), the anti-phospho-ERK1/2 (1:2000, Cell Signaling Technology, Danvers, CO, USA), and to normalize the protein amount loaded in the gel, the anti-β-Actin (1:10,000, Wuhan Fine Biotech Co., Dublin, CA, USA). Images were acquired with Azure C300 Processing machine (Azure Biosystem, Dublin, CA, USA). The intensity of bands was quantified with ImageJ software.

### 2.4. Over-Expression of miR-146a by Mimic Transfection

After hormones incubation, miR-146a mimic (Sigma Aldrich, Milan, Italy) was transfected at a concentration of 50 nM using Metafectene (Biointex, München, Germany), according to the manufacturer’s recommendations and incubated for 24 h. Cells incubated only with hormones served as negative controls and were set to 1 for analysis.

### 2.5. Statistics

All the results are reported as a mean value ± standard deviation (S.D.). Differences were analyzed with GraphPad Prism statistical program, using unpaired, two-tailed Student’s *t*-test. One asterisk, * *p* < 0.05; two asterisks, ** *p* < 0.01. For each type of experiment, a minimum of three independent biological replicates were performed. Normal distribution of data was tested using the Shapiro–Wilk test.

## 3. Results

### 3.1. miR-146a Directly Targets RNASEL 3′UTR Region in LM-20 and A375 Melanoma Cells and in HaCaT Keratinocytes

To evaluate the silencing role of miR-146a by targeting the consensus sequence in the 3′UTR region of the RNASEL messenger, we performed a luciferase transfection assay. Recombinant plasmids including a fragment of 777bp of RNASEL 3′UTR region containing a putative site for miR-146a binding (wild type, WT), as well as its mutated sequence (MUT), were cloned.

**Figure 1:**
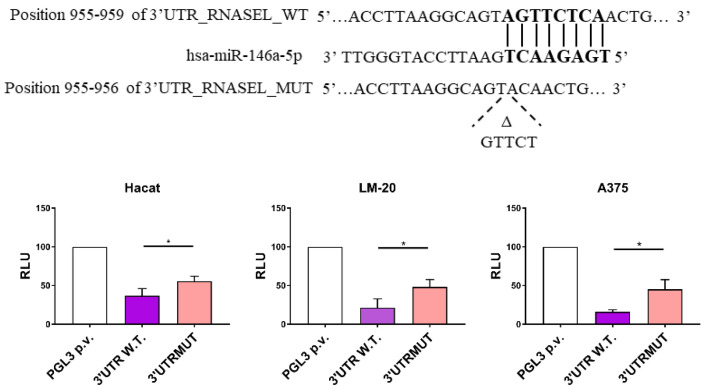
miR-146a-5p (miR-146a) directly regulates firefly luciferase activity through the ribonuclease L (RNASEL) 3′ untranslated regions (3′UTR). (**Top**) schematic representation of the miR-146a targeting sequence located in the 3′UTR of the RNASEL gene. (**Bottom**) firefly luciferase assay in HaCaT, LM-20 and A375 cells, respectively. All the results are reported as a mean value ± S.D. Differences in relative expression were analyzed with GraphPad Prism statistical program, using unpaired, two-tailed Student’s *t* test: * *p* < 0.05.

LM-20, A375 and HaCaT cell lines showed a significant down-regulation in the relative luciferase reporter activity when transfecting 3′UTR_RNASEL_WT construct in the presence of 50 nM miR-146a mimic. The corresponding mutated plasmid 3′UTR_RNASEL_MUT restored in part the activity of the luciferase (Figure 1). Thus, the results show that RNASEL mRNA is a target of miR-146a in the analyzed cell lines.

### 3.2. Effects of miR-146a Overexpression in the Presence of Hormones on RNASEL Gene Expression, RNase-L Protein Level and ERK1/2 Phosphorylation

**Figure 2:**
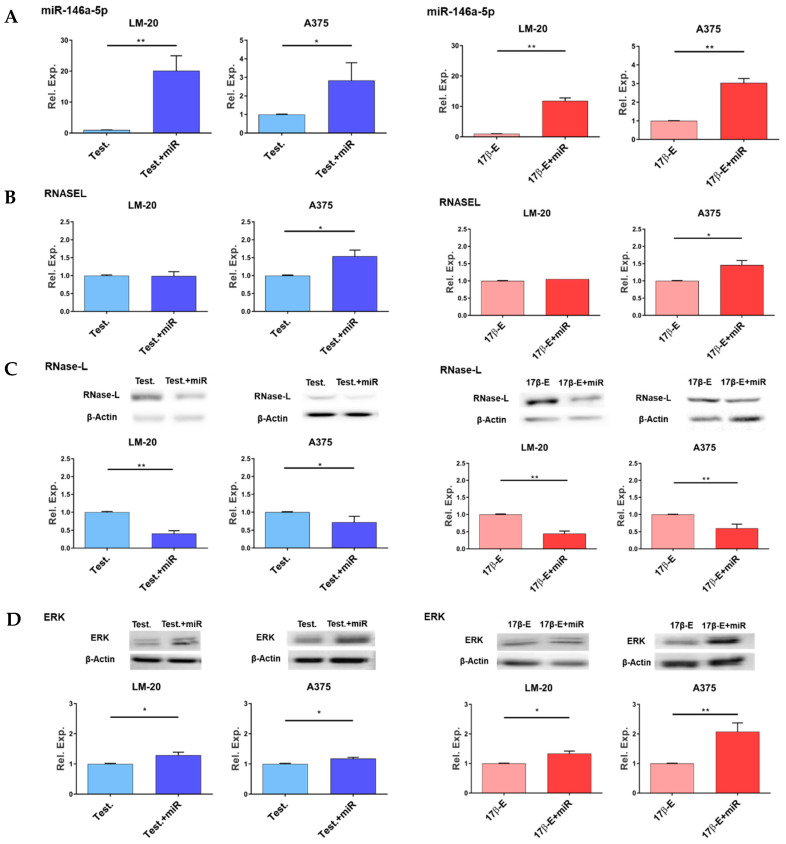
Effects of miR-146a mimic transfection on RNASEL gene, RNase-L protein, and phosphorylated extracellular signal-regulated kinases (pERK)1/2 expression levels in the presence of hormones. (**A**) Increase of miR-146a gene expression after miR-146a mimic transfection in the presence of either testosterone or 17β-estradiol in comparison with negative controls; (**B**, left) RNASEL transcript level in the presence of testosterone with (blue) or without (light blue) miR-146a mimic. (**B**, right) RNASEL transcript level in the presence of 17β-estradiol with (red) or without (pink) miR-146a mimic. (**C**, left) RNase-L protein level in the presence of testosterone with (blue) or without (light blue) miR-146a mimic. (**C**, right) RNase-L protein level in the presence of 17β-estradiol with (red) or without (pink) miR-146a mimic; (**D**) measure of the amount of the phosphorylated form of kinases ERK1/2 in the presence of each hormone. All the results are reported as a mean value ± S.D. Differences in relative expression were analyzed with GraphPad Prism statistical program, using unpaired, two-tailed Student’s *t*-test: * *p* < 0.05; ** *p* < 0.01.

Firstly, as a transfection efficiency control, the level of miR-146a expression in cells transfected with miR-146a mimic has been measured. Figure 2A shows higher miR-146 amount in cells transfected with miR-146a mimic in comparison with each negative control in both melanoma cell lines.

Overexpression of miR-146a in LM-20 cells did not affect the expression of RNASEL gene in the presence of each hormone. (Figure 2B, left). Conversely, miR-146a mimic transfection slightly increased RNASEL gene expression level in A375 cells in the presence of testosterone or 17β-estradiol (Figure 2B, right).

As expected, miR-146a overexpression resulted in a lower RNase-L protein amount in both cell lines cultured in the presence of testosterone, as well as in the presence of 17β-estradiol (Figure 2C).

To further demonstrate a pro-tumorigenic effect of miR-146a overexpression in melanoma cells, immunoblots were performed to measure the activated form of ERK1/2 enzymes. As shown in Figure 2D, miR-146a mimic transfection significantly increases the phosphorylated and active form of ERK1/2 in both A375 and LM-20 melanoma cell lines.

### 3.3. Sex Hormones Affect the Endogenous Expression of miR-146a and RNASEL Genes and RNase-L Protein in Melanoma Cells

Once the functional role of miR-146a on RNASEL/RNase-L expression has been validated by transfection experiments, we investigated the effect of the hormonal milieu on RNASEL, miR-146a, and RNase-L expression directly in cells. Melanoma cells were incubated with testosterone or 17β-estradiol for 24 h and subsequently the expression levels of miR-146a, RNASEL and RNase-L were evaluated by qPCR or Western Blot analyses.

#### 3.3.1. Effects of Hormones on RNASEL Gene Expression

**Figure 3:**
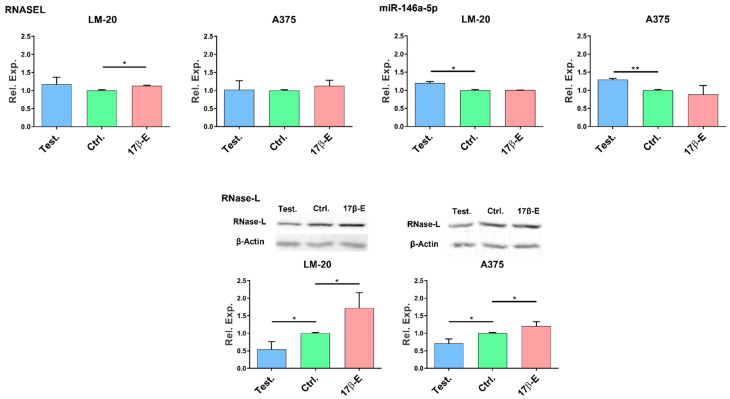
Sex hormones affect the endogenous expression of miR-146a, RNASEL, and RNase-L. In LM-20 and A375 cells: (**Top**, left panel) RNASEL transcript level in the presence of testosterone or 17β-estradiol in comparison with control. (**Top**, right panel) miR-146a gene expression level in the presence of testosterone or 17β-estradiol. (**Bottom**) RNase-L protein expression level in the presence of testosterone or 17β-estradiol. All the results are reported as a mean value ± S.D. Differences in relative expression were analyzed with GraphPad Prism statistical program, using unpaired, two-tailed Student’s *t*-test: * *p* < 0.05; ** *p* < 0.01.

The transcriptional level of RNASEL gene was measured by qPCR. In both melanoma cells, the presence of testosterone showed no significant effect on RNASEL gene expression. In contrast, 17β-estradiol increased RNASEL mRNA level in LM-20, thus supporting a transcriptional induction of the gene (Figure 3, Top, left panel).

#### 3.3.2. Effects of Hormones on miR-146a Gene Expression

The incubation with 17β-estradiol did not modulate miR-146a gene expression in either LM-20 nor in A375 cell lines. Conversely, testosterone induced a significant upregulation of miR-146a transcript in both LM-20 and A375 melanoma cell lines (Figure 3, Top, right panel).

#### 3.3.3. Effects of Hormones on RNase-L Protein Expression

The effects of sex hormones on the two different human melanoma cells were investigated by analyzing the expression level of RNase-L protein by western blot.

The expression of RNase-L was significantly diminished in both LM-20 and A375 melanoma cells (Figure 3, Bottom). On the contrary, 17β-estradiol administration increased the RNase-L protein amount in both melanoma cell lines (Figure 3, Bottom).

## 4. Discussion

Gene expression analysis in different non-reproductive tissues shows the presence of a gender-specific effect on gene transcription [43], and on gene epigenetic regulation [44]. Accumulating evidence demonstrate the advantages of women in various types of cancer, including colorectal cancer, urothelial, and kidney cancer [45,46], as well as melanoma [47]. In melanoma, differences between males and females in tumor thickness, anatomic location, and level of invasion are some of the factors that have been linked to the progression of malignancy and survival of patients suggesting explanations for the female melanoma advantage [48,49,50,51]. Sex hormone signaling can affect cancer predisposition through several mechanisms, influencing tumor microenvironment, immune system, and the overall metabolic balance of an organism [8,9,10,11,48].

We had previously shown that polymorphisms of RNASEL and miR-146a genes were associated with melanoma in a sex-specific manner [13]. The present work intended to investigate, in sex-hormone-treated melanoma cell lines, the contribution of RNASEL, its product RNase-L enzyme, and miR-146a in the female advantages observed in melanoma incidence and survival.

It is well known that RNASEL 3′UTR region displays a putative binding site for miR-146a [31]; however, the functionality of this site was not proven so far. At the present point, we attest that RNASEL mRNA is a target of miR-146a since the binding of miR-146a to RNASEL 3′UTR region lowers the luciferase reporter activity in two melanoma cell lines and non-transformed keratinocytes, as well (Figure 1).

Moreover, overexpression of miR-146a by mimic transfection in the presence of either testosterone or 17β-estradiol can decrease RNase-L protein expression without interfering with RNASEL mRNA level in LM-20 melanoma cells (Figure 2). This suggests that miR-146a was able to decrease RNase-L protein amount likely by binding to RNASEL transcript and destabilizing its translation. Dissimilar results were obtained with miR-146a mimic transfection in A375 melanoma cells after treatment with each hormone, in which RNASEL transcript level was slightly upregulated (Figure 2B). This unexpected data could be due to an indirect downregulation of a miR-146a-target repressor factor acting on RNASEL gene promoter or by a direct activation performed by miR-146a itself on RNASEL gene transcription, as recently described [52]. In any case, this result needs further insights in the future. Nevertheless, at the protein level in the presence of each hormone, miR-146a mimic drives a slight but significant decrease in the amount of RNase-L enzyme, suggesting that the inhibitory effect of miR-146a could be dominant on RNASEL transcriptional activation (Figure 2C). In cancer, through different signaling pathways, miR-146a can affect the expression of several cancer-related genes and ultimately have dual conflicting roles in tumor progression and metastasis [25]. In fact, miR-146a displayed tumor suppressive functions in pancreatic cancer cells and colon cancer [53,54]. Conversely, in melanoma, miR-146a plays an oncogenic role. Indeed, it is upregulated by the oncogenic kinases BRAF and NRAS, and its overexpression in melanoma cells promotes proliferation and leads to tumor formation when overexpressing cells are injected in mice [26,55]. In concert, our data showed an increase in the phosphorylation level of the ERK1/2 MAPK in both melanoma cell lines transfected with miR-146a mimic (Figure 2C). Thus, miR-146a overexpression, in sex-hormones treated melanoma cells, supports the oncogenic role of miR-146a in melanoma by downregulating RNase-L expression and by activating the growth-promoting kinases ERK1/2.

Subsequently, we investigated in the context of melanoma cells whether testosterone or 17β-estradiol administration may affect the endogenous expression of RNASEL and miR-146a genes and RNase-L protein.

Our data attest that the expression of miR-146a was significantly increased by testosterone in two melanoma cell lines (Figure 3). The augmented expression of miR-146a may represent a disadvantage in males since it can act as a negative regulator of immune activation and can negatively interfere, among others, with the signal transduction and activator of transcription (STAT)-1/interferon (IFN)-γ axis, which in turn can reduce cell-migration, cell cycle activity, and basal oxygen consumption rate in melanoma cells [56,57]. Furthermore, miR-146a promoted primary melanoma progression by activating Notch signaling as described by Forloni et al. [26].

RNASEL gene expression in the presence of either hormone displayed only little difference compared to untreated control cells. A very slight but significant increase in RNASEL transcript was registered only with 17β-estradiol treatment in LM-20 cells (Figure 3). The majority of variations emerged when RNase-L protein expression was assessed. We observed in both melanoma cell lines an increased amount of RNase-L protein after cell incubation with 17β-estradiol and a lower enzyme expression with testosterone treatment (Figure 3). We can speculate that a lower RNase-L protein amount caused by testosterone could result from a post-transcriptional downregulation of the gene that could be driven, at least in part, by the concomitant upregulation of miR-146a. Whereas, the higher expression of the enzyme in 17β-estradiol-treated cells could be a consequence of transcriptional RNASEL induction.

For its endo-ribonucleolytic activity, RNase-L regulates cell growth and differentiation by cleaving and destabilizing several mammalian RNA targets [58]. Notably, RNase-L targets include many miRNAs transcripts as well. Thereby, RNase-L can act as tumor-suppressor in mammalian cells also via destabilization of the miRNA-regulated transcriptome [58]. This multiplicity of targets, which is a typical feature of other antitumor RNases [59], results in pleiotropic effects, ranging from cell growth inhibition to pro-apoptotic activity [57], and suggests that RNase-L can play a protective effect on tumor progression and participate in the survival advantage in female melanoma patients. Remarkably, RNase-L negatively regulates androgen signaling by a protein-protein interaction mechanism [60]. Again, the high level of RNase-L expression in the presence of 17β-estradiol could result in further suppression of androgenic response; conversely, the low expression level in testosterone-treated cells can further potentiate AR signaling. Androgen signaling, indeed, has been demonstrated to promote cancer progression by inhibiting apoptosis or by promoting cell migration and matrix metalloproteinase activity in prostate cancer cells [60].

As previously reported [10], the major committed for the worse melanoma prognosis of males versus females could be the higher level of testosterone, which in turn activates AR. Genetic or pharmacological suppression of AR activity in melanoma cells hinders their proliferation [10]. Very recently, Vellano et al. demonstrated that AR blockage promoted a better response to BRAF/MEK-targeted therapy in melanoma patients, thus improving recurrence-free survival [61].

## 5. Conclusions

In conclusion, the results obtained in this study suggest that sex hormones may act on miR-146a, on RNASEL gene expression, and especially on RNase-L protein level with a view to contribute to the female advantages observed in melanoma incidence and survival.

## Figures and Tables

**Table 1:** Primers and probes used for Real-Time PCR and primers used for plasmid construction.

Genes	Primers Sequences	Products
Real-Time PCR (mRNA)	Primers (5′ → 3′)	Product (bp)
RNASEL	CAGGATCTGCAACCACAAAA	82
	CCCACTTGATGCTCTTATCAAA	
TBP	TGTATCCACAGTGAATCTTGG	102
	ATGATTACCGCAGCAAACC	
Real-Time PCR (microRNA)	Sequences (Probe)	Assay (ID)
hsa-miR-146a-5p	UGAGAACUGAAUUCCAUGGGUU	478399_mir
hsa-miR-191-5p	CAACGGAAUCCCAAAAGCAGCUG	478231_mir
Plasmid construction	Primers (5′ → 3′)	Product (bp)
3′UTR RNASEL_W.T.	CCTTCTAGAAACAAGCCTCAGTGTGATGG	1771
	GGATCTAGACCAGGTGCTCATTACAAATC	
^(−)^3′UTR RNASEL_W.T.	CCTTCTAGAAACAAGCCTCAGTGTGATGG	777
	GGATCTAGAACCAAAAACTTCTTCAGACTC	
3′UTR _MUTAGENESIS	ATGACCTTAAGGCAGTACAACTGGGGGGCAATTT	
	AAATTGCCCCCCAGTTGTACTGCCTTAAGGTCAT	

## Data Availability

Not applicable.
